# Silica nanoparticles-induced cytotoxicity and genotoxicity in A549 cell lines

**DOI:** 10.1038/s41598-024-65333-5

**Published:** 2024-06-24

**Authors:** Zahra Peivandi, Farshad H. Shirazi, Shahram Teimourian, Golrokh Farnam, Vahid Babaei, Neda Mehrparvar, Nasim Koohsari, Azadeh Ashtarinezhad

**Affiliations:** 1 Department of Occupational Health Engineering, School of Public Health, Iran University of Medical Sciences, Tehran, Iran; 2 Pharmaceutical Sciences Research Center, Shahid Beheshti University of Medical Sciences, Tehran, Iran; 3 Department of Pharmacology/Toxicology, School of Pharmacy, Shahid Beheshti University of Medical Sciences, Tehran, Iran; 4 Department of Medical Genetics, School of Medicine, Iran University of Medical Sciences, Tehran, Iran

**Keywords:** Lung cancer, Biomarkers, Gene regulation, Risk factors

## Abstract

Among the myriad of nanoparticles, silica nanoparticles (SiO_2_NPs) have gained significant attention since they are extensively produced and used across several kinds of industries. Because of its widespread usage, there has been increasing concern about the potential health effects. This study aims to evaluate the effects of SiO_2_NPs on Interleukin-6 (IL-6) gene expression in human lung epithelial cell lines (A549). In this study, A549 cells were exposed to SiO_2_NPs at concentrations of 0, 1, 10, 50, 100, and 200 µg/mL for 24 and 48 h. The IL-6 gene expression was assessed using Real-Time RT-PCR. Additionally, the impact of SiO_2_NPs on the viability of A549 cells was determined by MTT assay. Statistical analysis was performed using GraphPad Prism software 8.0. MTT assay results indicated a concentration-dependent impact on cell survival. After 24 h, survival decreased from 80 to 68% (1–100 µg/mL), rising to 77% at higher concentrations. After 48 h, survival dropped from 97 to 80%, decreasing to 90% at higher concentrations. RT-PCR showed a dose–response relationship in cellular toxicity up to 10 µg/mL. At higher concentrations, there was increased IL-6 gene expression, mitigating SiO_2_NP-induced cytotoxic effects. The study shows that the viability and proliferation of A549 cells are impacted by different SiO_2_NPs concentrations. There may be a potential correlation between IL-6 gene expression reduction and a mechanism linked to cellular toxicity. However, at higher concentrations, an unknown mechanism increases IL-6 gene expression, reducing SiO_2_NPs' cytotoxic effects. These effects are concentration-dependent and not influenced by exposure times. Further investigation is recommended to determine this mechanism's nature and implications, particularly in cancer research.

## Introduction

The rapid advancement of nanotechnology and the growing incorporation of silica nanoparticles (SiO_2_NPs) into a multitude of sectors, such as electronics^1^, medicine^2^, food industry^3^, and energy production^4^, has prompted a surge in interest in these nanoparticles^5^, particularly concerning their potential implications for human health and the environment^6,7^. SiO_2_NPs characterized by their unique properties, have been directly integrated into an array of products, thereby driving the expansion of the SiO_2_NPs industry^8^. Moreover, the synthesis of SiO_2_NPs holds significant implications for the advancement of nanomedicine, offering opportunities for tailored drug delivery systems and imaging agents due to their inherent biocompatibility and tunable surface properties^9,10^. However, the health and ecological implications of SiO_2_NPs remain largely unexplored^11^, with incomplete information regarding their storage and utilization^12^. Research has demonstrated that SiO_2_NPs can infiltrate the human body via various pathways, including inhalation, ingestion, skin contact, and injection^13^. The toxicological mechanisms underlying SiO_2_NPs are not yet fully understood, yet they are implicated in oxidative stress, endoplasmic reticulum stress, lysosomal barrier dysfunction, mitochondrial dysfunction, and other factors^14^. The detrimental health effects attributed to SiO_2_NPs encompass alterations in bodily functions, fibrosis, cardiac energy metabolism, and potential neurotoxicity^15^. Research indicates that SiO_2_NPs can disrupt hematological homeostasis by promoting megakaryocyte development and platelet formation^16^, induce ferroptosis in human umbilical vein endothelial cells, potentially affecting cardiovascular health^17,18^, aggravate metabolic associated fatty liver disease (MAFLD) progression by promoting hepatic steatosis and oxidative stress, and trigger inflammatory responses and ferroptosis in neuroimmune cells, leading to neurotoxicity^14,19^. These findings highlight the diverse toxicological effects of SiO_2_NPs on different organ systems, emphasizing the importance of understanding and mitigating the potential risks associated with their widespread use in various industries^14^.

SiO_2_NPs have been demonstrated to induce adverse signaling in a range of cells, including endothelial cells, macrophages, and lung cancerous cells, thereby establishing common pathways and activating stress responses^20^. In the field of nanotoxicology, initial screening of nanoparticle toxicology research typically employs laboratory evaluation methods using cell cultures^21^. Various in vitro assays, such as cell viability assessments, oxidative stress assays, and particle size analyses, are utilized to evaluate nanoparticles' effects on cells^22^. Additionally, while in vivo studies are essential for a comprehensive understanding of nanomaterial toxicity, in vitro methods serve as the foundational evaluation to assess potential risks^23^. Efforts are ongoing to standardize in vivo nanotoxicology studies and enhance the precision of in vitro methods, which are crucial for advancing the safety profile of nanomaterials^24^. Recent studies have examined the toxicity of SiO_2_NPs on HepG2 cell lines, revealing that SiO_2_NPs can induce apoptosis and autophagy, leading to cytotoxicity and oxidative stress in cells. These findings underscore the importance of continued research into the health effects of SiO_2_NPs, with a focus on understanding their interactions with cellular reactive oxygen species and glutathione, as well as the activation of cell death in response to SiO_2_NPs exposure^25^. Research has demonstrated that exposure to crystalline SiO_2_NPs and microparticles leads to a decrease in cell survival, an increase in reactive oxygen species (ROS) production, mitochondrial membrane damage, and a reduction in the antioxidant content of cells in a concentration- and time-dependent manner^5^. The observed effects of SiO_2_NPs on the viability of A549 cells can be influenced by a multitude of factors. For instance, a study conducted by Brown et al. revealed that the pro-apoptotic effect of SiO_2_NPs is contingent upon their size and dose, as well as the specific type of glioblastoma cells. It is suggested that these nanoparticles exert a significant impact on the survival of A549 cells by inducing oxidative stress and apoptosis^26^. On the other hand, at the intersection of nanoparticle toxicity and inflammatory responses lies interleukin 6 (IL-6), a pivotal cytokine with multifaceted roles in immunity and inflammation and is involved in a variety of cellular processes including differentiation, proliferation, and apoptosis^27,28^. Interleukin-6 (IL-6) is an important cytokine in adaptive immune responses, as well as in infection, autoimmune disorders, cardiovascular diseases, and certain cancers. Examining the various effects observed by researchers could potentially indicate the pathological conditions associated with IL-6, ranging from inhibiting cell growth to promoting cell proliferation. Therefore, our focus has been on studying the expression of this protein in cells following exposure to nanoparticles.

IL-6 is produced by various cell types, including macrophages, fibroblasts, and endothelial cells, in response to a wide range of stimuli, including infection, tissue damage, and inflammation^29^. It is known to be a key mediator in the acute phase response and is often upregulated in response to infections and tissue injuries^30^. Elevated levels of IL-6 have been implicated in chronic inflammation and various inflammatory diseases, making it a significant marker for studying the inflammatory and immune responses in cells^31^. Understanding the interplay between IL-6 and nanoparticle toxicity is essential for elucidating the mechanisms underlying nanoparticle-induced inflammation and its implications for human health. Therefore, this study aims to evaluate the expression of the interleukin 6 gene on human lung cancerous epithelial cells (A549) induced by SiO_2_NPs, to understand the cellular and molecular mechanisms underlying the potential toxicity of these nanoparticles and identify potential respiratory hazards for exposed workers.

## Method and materials

### SiO_2_ nanoparticles

Silicon Dioxide Nanopowder (SiO_2_, 99+%, 20–30 nm, amorphous) was purchased from the US Research Nanomaterials, Inc. company (Stock: 3438, CAS: 7631-86-9).

### Silica nanoparticles preparation

The sanitization process was done first to ensure the absence of biological interferences in cell viability. In this study, 4 mg of nanoparticles were weighed and then autoclaved using an autoclave (Model: K.T.9.100-2, Reyhan Teb, Iran) at 121 °C for 25 min. The autoclaved nanoparticles were then placed under the hood and dissolved in 2 ml of DMEM culture media. After the sterilization process, SiO_2_NPs were diluted with concentrations of 1, 10, 50, 100, 150, and 200 μg/ml, and then sonicated using an ultrasonic device (Elma Ultrasonic, Type: S60H, Germany) for 30 min.

### Cellular section (cell culture and cytotoxicity assessment)

#### Cell culture and cell seeding

A549 (lung adenocarcinoma epithelial cell line) was obtained from the National Cell Bank of Iran (NCBI, Pasteur Institute of Iran, Tehran, Iran). Cells were cultured in DMEM (Dulbecco’s Modified Eagle Medium) growth medium for routine culture supplemented with 10% FBS (Fetal bovine serum), and 1% pen/strep antibiotic mixture solution. Growth conditions were maintained at 37 °C, 5% CO, and 95% humidity and subcultured by trypsinization followed by splitting the cell suspension into fresh flasks and supplementing with fresh cell growth medium. Before the start of the experiment, the growth medium of near-confluent cells was replaced with fresh media changed every three days. DMEM Medium, FBS, Pen-strep, and Trypsin EDTA 1× were purchased from Gibco (USA).

#### Cell viability assessment by MTT assay

The cytotoxicity of SiO_2_NPs was evaluated using MTT assay. order to examine the biological effect of SiO_2_NPs, The A549 cells were seeded at a density of 3 × 10^3^ cells per well in 96-well plates and were grown for 24 h. The exponentially growing A549 cells (approximately 80% confluence) were exposed to SiO_2_NPs’ concentrations of 0, 1, 10, 50, 100, and 200 µg/mL in triplicates for the exposure time (incubation) of 6, 12, 24, and 48 h. Media without SiO_2_NPs was added to the control wells. At the end of the incubation period, 20 µl of 3-[4,5-DimethyIthiazol-2-yl]-2,5-diphenyl tetrazolium bromide (MTT) procured at the concentration of 5 mg/ml in 1× PBS was added to each well. The plates were incubated in a CO_2_ incubator at 37 °C for 3 h. After that, the MTT solution was removed and Formazan crystals were dissolved by adding 100 ml of DMSO per well at 37 °C. After 20 min of incubation in dark conditions on a shaker. The absorbance was measured using a 96-well plate reader (ELISA-Reader BioTek). The viability of the cell was calculated using the flowing formula:

$${\text{Cell Viability }}\left( \% \right) \, = \, \left( {\text{Absorbance of exposed cells/Absorbance of control cells}} \right) \, \times { 1}00$$

### Molecular section

#### Sample preparation for RNA extraction

A549 cells were cultured in 6-well plates at a density of 3 × 10^5^ cells per well for 24 h. following this incubation, these cells were exposed to various concentrations of SiO_2_NPs (0, 1, 10, 50, 100, and 200 μg/ml) for 24 and 48 h. In order to prepare the samples for RNA isolation, the culture media was removed, and then the cells were washed with normal saline before being treated with trypsin. After adding the culture medium and pipetting, the suspensions inside each well were transferred to the microtube. After centrifugation, the supernatant was discarded and cell pellets were collected at the bottom of the microtubes. for cell storage, pelleted cells were resuspended in a small amount of PBS then 5 volumes of RNA Later (Yekta Tajhiz Azuma, cat: YT9085) solution was added to each microtube, and the samples were placed in a freezer at – 80 °C for RNA analysis.

#### RNA extraction

In this study, we employed the SINACLON RNX-Plus Solution (RNA isolation kit protocol, SINACLON, cat.NO:EX6101) for the isolation of total RNA from samples) Reagents: Chloroform, Isopropanol, 75% Ethanol, DEPC treated water(. The protocol involved several steps. In summary, the cells were washed with PBS. Subsequently, 1 ml of chilled RNX-Plus solution was added to each sample and incubated at room temperature for 5 min. To facilitate cell lysis, 200 μl of chloroform was added and the mixture was incubated at 4 °C for 5 min. The resulting mixture was then centrifuged, and the aqueous phase containing RNA was carefully transferred to a new microtube. An equal volume of isopropanol was added to the aqueous phase to precipitate the RNA. After 15 min of incubation on ice, a second centrifugation step was performed at 4 °C for 15 min to pellet the RNA. The resulting pellet was washed with 1 ml of 75% ethanol and centrifuged at 7500*g* and 4 °C for 10 min. Finally, the pellet was resuspended in DEPC-treated water after partial drying. To aid in the dissolution process, the samples were incubated in a water bath at 60–55 °C for 10 min. The quantity of the extracted RNA was assessed by measuring the absorbance at 260 and 280 nm using a NanoDrop Thermo 2000 Spectrophotometer (cat.NO:ND-2000, USA) for subsequent cDNA synthesis.

#### cDNA synthesis

For cDNA synthesis, the EasyTM cDNA Synthesis Kit from Parstous was utilized. The kit includes Buffer-Mix (*2), Enzyme-Mix, and DEPC-treated Water. Following the quantification of RNA concentrations using a NanoDrop (Thermo 2000 Spectrophotometer, cat.NO:ND-2000,USA), the samples were normalized and transferred to labeled microtubes. Then, 10 µl of Buffer Mix (*2) and 2 µl of Enzyme Mix were added to each microtube. Then, each sample was brought to a final volume of 20 µl using DEPC-treated water. It is recommended to add the enzyme in the final step. The samples were then subjected to a thermal cycler (BIO-RAD T100-THERMAL CYCLER) according to the protocol. The reaction protocol was followed: priming at 25 °C for 5 min, reverse transcription at 47 °C for 60 min, termination of the reaction at 85 °C for 5 min, and finally, holding the reaction by Chilling on the ice at 4 °C. The synthesized cDNA was stored at − 20 °C until it was used for real-time RT-PCR. All the reactions were performed in duplicate to ensure reproducibility of the results.

### Primer design and PCR

The primer sequences used in this research for IL-6 were as follows: forward primer (GGTACATCCTCGACGGCATC) and reverse primer (CACCAGGCAAGTCTCCTCATT). These primers were designed using the Primer3plus software.

### RT-PCR analysis and qualitative assessment of interleukin-6 gene

To perform RT-PCR, the following protocol was followed: a final volume of 20 μl was prepared. The components included 10 µl of the Master Mix PCR amplicon, 0.5 µl of the forward primer (PF), 0.5 µl of the reverse primer (PR), 3 µl of cDNA, and 6 µl of DNase-free water. The mixture was thoroughly mixed. The RT-PCR was carried out using the Bio-Rad T100-Thermal Cycler device. The following temperature and time program was used: an initial denaturation step at 95 °C for 3 min in the first cycle, followed by 35 cycles of denaturation at 94 °C for 30 s, annealing at 60 °C for 30 s, and extension at 72 °C for 5 min. A final extension step was performed at 72 °C for 5 min. Following that, a conventional Polymerase Chain Reaction (PCR) was conducted for a qualitative analysis of the Interleukin-6 gene. In this step, the samples were first run at 80 amperes for 5 min and then run at 120 amperes for 25 min on a 1.5% agarose gel in 10 μl volumes. This step, known as electrophoresis, separates DNA bands of various sizes within the gel.

### Real-Time RT-PCR analysis and quantitative assessment of interleukin-6 gene

The Interleukin-6 gene was selected as the target gene, and GAPDH was chosen as the reference gene. Subsequently, Real-time RT-PCR reactions for the target and reference genes were performed on the A549 cell line as follows: Real-time RT-PCR was conducted using the Light Cycler TM 96 device (Roche, Germany) with the following temperature and time program: Initially, a cycle at 95 °C for 3 min was performed, followed by the following temperature cycling in 35 repeated cycles: 94 °C for 30 s, 60 °C for 30 s, a final extension at 72 °C for 30 s, and 72 °C for 5 min. The reaction was repeated twice for each cDNA sample, and Ct values were calculated. Changes in mRNA expression were quantified using the relative gene quantification method (2^−ΔΔCT^ method).

### Statistical analysis

Statistical analysis was performed using GraphPad Prism software 8.0 (GraphPad Software, Inc., San Diego, CA, USA) and SPSS (version 18.0; SPSS, Inc., Chicago, IL, USA). All experiments were performed in triplicate and data were expressed as the means ± standard deviation. A p-value less than 0.05 (≤ 0.05) was considered statistically significant.

## Results

### Cytotoxicity of SiO_2_NPs by MTT assay

**Figure 1:**
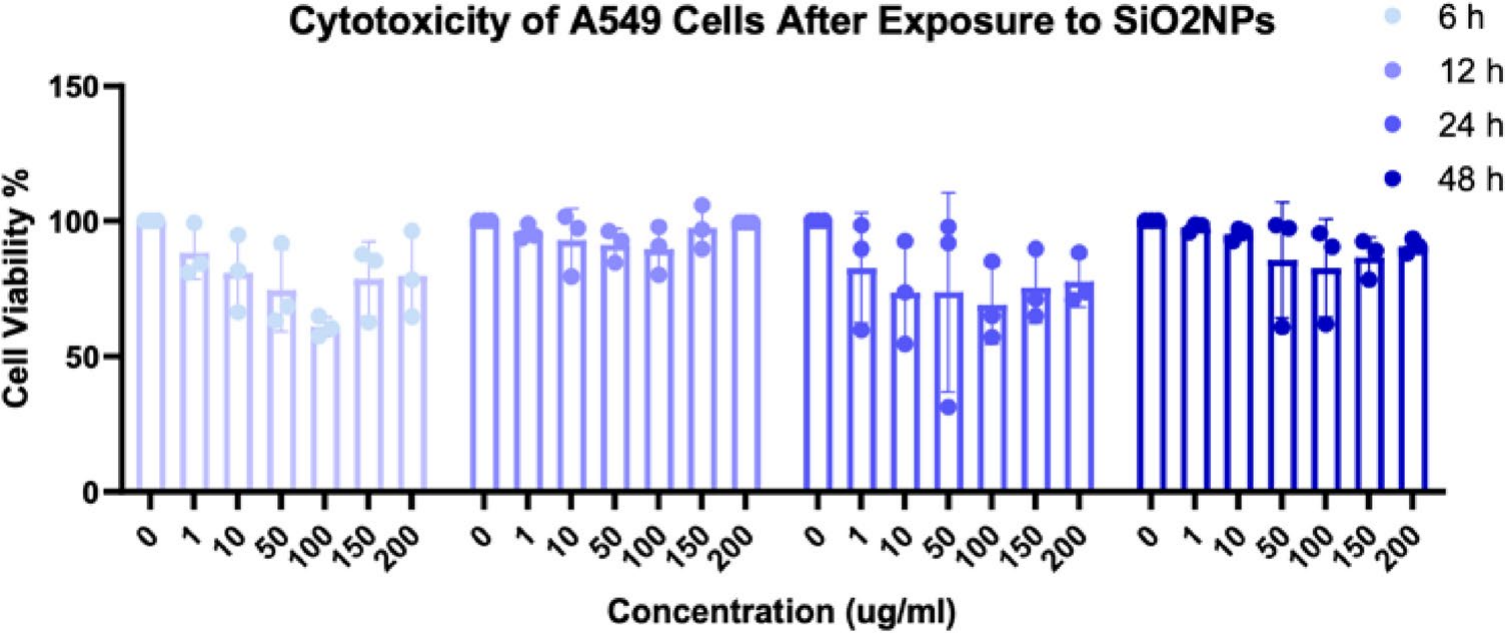
Comparison of A549 cells viability percentage after 6, 12, 24, and 48 h of exposure (all results were performed in triplicate, and data were expressed as the means ± standard deviation. A p-value less than 0.05 (≤ 0.05) was considered statistically significant (Table 1).

The investigation of the toxicity of nanoparticles and the related tests were done after the fifth passage. The MTT assay results showed that cell viability and growth were affected by different concentrations of SiO_2_NPs. Exposure to SiO_2_NPs led to a time-dependent decrease in the survival of A549 cells. However, with increasing nano concentration, cell growth increased gradually (Fig. 1). The findings revealed a statistically significant difference (p-value < 0.05) in the cell viability percentage after exposure to different time intervals (6 and 12 h compared to 12 and 48 h), as detailed in Table 1.

**Table 1:** Comparison of A549 cells viability percentage after exposure for 6, 12, 24 and 48 h.

Tukey's multiple comparison test	Mean diff.	95.00% Cl of diff.	Significant?	Summary	Adjusted P value
6 h vs. 12 h	− 14.77	− 26.89 to − 2.647	Yes	*	0.0216
6 h vs. 24 h	1.570	− 4.928 to 8.068	No	ns	0.8359
6 h vs. 48 h	− 10.75	− 19.30 to − 2.206	Yes	*	0.0187
12 h vs. 24 h	16.34	6.092 to 26.59	Yes	**	0.0060
12 h vs. 48 h	4.015	− 2.986 to 11.02	No	ns	0.2899
24 h vs. 48 h	− 12.32	− 20.71 to − 3938	Yes	**	0.0090

*P ≤ 0.05, **P ≤ 0.01.

### RNA isolation quantification

After culturing the A549 cells, the required RNAs were extracted and their concentrations were evaluated. The absorbance ratio at 260 to 280 nm was determined to be 1.8 or less during the NanoDrop test, demonstrating an acceptable level of RNA purity in the samples. Therefore, the results indicate that the extracted RNA could be used effectively in the following step of this study (cDNA synthesis).

### Interleukin-6 gene quality assessment

Following cDNA synthesis, PCR was used to evaluate the quality of the interleukin-6 gene, and samples were run on a 1.5% agarose gel. To identify the target gene band on the electrophoresis gel, a ladder marker (M) was utilized as a reference. The required band with a length of 167 base pairs (bp) was found based on the electrophoresis results, showing the presence or expression of the interleukin-6 gene in this band area, or in other words, in the A549 cells.

### Determination of interleukin-6 gene expression using real-time RT-PCR method

**Figure 2:**
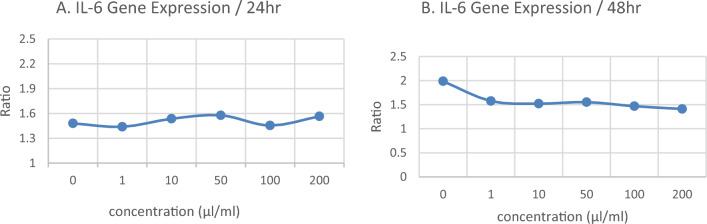
The level of interleukin-6 gene expression after exposure for 24 (**A**) and 48 (**B**) hours.

The results showed that the interleukin-6 gene is expressed in the A549 cell line. This test was performed after 24 and 48 h of exposure to SiO_2_NPs at concentrations of 1, 10, 50, 100, and 200 μg/ml. The results indicate that the cytotoxicity of SiO_2_NPs and their effects were concentration-dependent and not dependent on the exposure time to the SiO_2_NPs (Fig. 2).

## Discussion

SiO_2_NPs have been shown to have adverse effects on the respiratory system^32^. In this study, we evaluated the expression of the Interleukin 6 gene on human lung cancerous epithelial cells (A549) induced by SiO_2_NPs. The MTT assay was conducted on exposed A549 cells to SiO_2_NPs at concentrations of 0, 1, 10, 50, 100, 150, and 200 µg/ml for 6, 12, 24, and 48 h. The results showed that cell viability increases at concentrations higher than 100 μg/ml compared to lower concentrations. The results of the MTT test showed that with the increase in the concentration of SiO_2_NPs after exposure for 6 h from 1 to 100 μg/ml, the percentage of cell survival decreased from 87 to 60% and caused cell death. But in concentrations higher than 100–200 μg/ml, the cytotoxicity effects of SiO_2_NPs disappeared and the percentage of cell survival increased from 60 to 78%. The survival percentage of cells decreased from 95 to 89% after 12 h in concentrations of 1 to 100 µg/ml and increased to 99% in higher concentrations. Moreover, the cell survival percentage at concentrations of 1 to 100 μg/ml decreased from 80 to 68% after 24 h of exposure and increased to 77% at higher concentrations. Also, 48-h exposure resulted in a decrease in cell survival percentage from 97 to 80% at concentrations of 1 to 100 μg/ml and an increase to 90% at higher concentrations (Fig. 1). The present study showed that the toxicity of SiO_2_NPs at low concentrations is greater than the exposure at higher amounts, which is similar to the study by Wu et al. in 2019, A549 cells were exposed for 12 and 24 h with different concentrations of 100, 500 and 1000 ng/ml. SiO_2_NPs exposure led to a time-dependent decrease in A549 cell viability. However, with the increase in the concentration of SiO_2_NPs, the cellular growth gradually returned and the survival of the cells increased^33^. Lin W and his colleagues in 2006, investigated the cytotoxicity of s SiO_2_NPs with a diameter of 25 nm and 46 nm on A549 cells. The results indicated that the level of toxicity is dose-dependent and increasing the concentration of nanoparticles decreases the survival of A549 cells^34^. The evaluation of the effects of SiO_2_NPs on the percentage of cell viability has also brought different results. Rafieipour et al. in 2023, found that crystalline SiO_2_ nanoparticles and microparticles reduce cell survival and reduce the antioxidant content of cells in a concentration- and time-dependent manner^5^. On the other hand, a study by Ahamed et al. in 2013 showed that SiO_2_ nanoparticles cause cytotoxicity and apoptosis in A549 cells. These findings suggest that SiO_2_NPs may have a significant effect on the viability of A549 cells, possibly through the induction of oxidative stress and apoptosis^35^. Also, Karimi et al.'s study in 2021 showed that the toxicity of SiO_2_NPs is dependent on the concentration of SiO_2_NPs and the cell type. As the concentration of SiO_2_NPs increases, the survival rate of cells decreases. Also, the toxicity of SiO_2_NPs against normal human lung cells was lower than human lung cancerous cells^36^. However, the observed effects of SiO_2_ nanoparticles on the viability of A549 cells can be influenced by various factors. For example, a study by Brown et al. in 2007 showed that the pro-apoptotic effect of SiO_2_NPs depends on their size and dose, as well as the type of glioblastoma cells^26^. Similarly, Lee et al., in 2019, in their study on the toxicity of SiO_2_NPs with a size of 10 nm in the concentration range of 5 to 15 μg/ml on human endothelial cells by MTT test, concluded that by increasing the concentration of SiO_2_NPs, the survival of endothelial cells decreases^37^. The results of Maqusood Ahamed's research in 2013 on the percentage of skin endothelial survival (A431), human lung epithelial cells (A549), and cells that were exposed to SiO_2_NPs with a size of 25 nm (concentration range of 15–100 μg/ml) also show that Cell survival has been decreasing in both types of cells^35^. On the other hand, Christian Chapa et al.’s study in 2014 indicated that surface modification impacts cell viability, with silica-coated magnetite nanoparticles exhibiting higher stability and cell viability compared to oleic acid-coated ones. Furthermore, the concentration of nanoparticles influences cytotoxicity, highlighting the importance of concentration in biomedical applications which underscores the significance of nanoparticle concentration in directing cell response and viability^38^. These conflicting findings suggest that the cytotoxicity of SiO_2_NPs on A549 cells is dependent on the particle concentration, but further research is needed to elucidate the underlying mechanisms and determine the exact dose–response relationship between SiO_2_NPs and A549 cell viability, as well as to fully understand the underlying mechanisms of these effects and their potential implications for the use of SiO_2_NPs are needed. In addition, it is important to consider the limitations of the exposure concentration and duration of the experiment in these studies, as they may have influenced the observed results.

Furthermore, based on the evaluation of the effects of SiO_2_NPs on IL-6 gene expression in cells (Fig. 2), the Real-time RT-PCR results showed a dose–response relationship in cellular toxicity in cells exposed to SiO_2_NPs, which is evident up to a concentration of 10 μg/ml. However, at higher concentrations, this mechanism led to an increase in IL-6 gene expression and a reduction in the cytotoxic effects of SiO_2_NPs. The difference between the two Real-Time RT-PCR graphs after 24 and 48 h of exposure did not reveal a time-dependent mechanism. Instead, it emphasized the concentration-dependent fluctuations of SiO_2_NPs caused by competitive and counteractive reactions with one another within the cell during the first 24 h after exposure for the expression of the interleukin-6 gene, which significantly reduces and increases the expression levels of this gene (Fig. 2). That is to say, after the SiO_2_NPs exposure, the IL6 gene is expressed inside the cell, which makes the expression of this gene very low and high. The measurement of IL6 gene expression after 48 h, during which the molecular mechanisms have gone through the initial fluctuations, confirms the predominance of counter-reactions and the stability of the expression of this gene in values similar to normal intracellular values. This is if the study by Jing Wu et al. in 2019 showed that SiO_2_NPs have a toxic effect on A549 cells and the expression of IL-1b in A549 increased, creating an inflammatory effect^33^. Also, Hoeta et al. in 2012, found in their experiment that in general, according to cytokine measurements in co-cultures of epithelial cells and macrophages, TNF-α, IL-8, and IL-6 are the most evident and significant secretion of all cytokines for 2 nm particles were observed^39^. The results of Xiao Jing Yang and colleagues in 2023, also showed that SiO_2_NPs cause toxic effects on cells and tumor necrosis factor TNF-α, interleukin-6 (IL-6) were expressed^40^. Moreover, the study conducted by Gualtieri in 2012, also showed that amorphous SiO_2_NPs at concentrations of 30 and 50 nm and rhodamine-coated SiO_2_NPs at a concentration of 50 nm induce pro-inflammatory responses and showed that SiO_2_NPs induce the expression of IL6 and IL8^41^. Therefore, the findings of this research are Intriguing and thought-provoking in the importance of counter and competitive reactions of dose-dependent cellular detoxification, and at the same time, more research is needed to identify the unknown mechanism that counteracts the effects of SiO_2_NPs toxicity, which also has the molecular effects of IL6 gene expression returned, are necessary.

## Conclusion

The study shows that the viability and proliferation of A549 cells are impacted by different SiO_2_NP concentrations. Cellular effects of various materials, such as nanoparticles, may not be fully comprehended through a cytotoxicity test like MTT. A deeper comprehension necessitates insights into the underlying mechanisms of cytotoxicity. By selecting IL-6 as a cytokine influencing both cell proliferation and suppression, it becomes evident that, despite the cytotoxic results, cells experience intricate feedback cascades over time. The variations in IL-6 expression following exposure to different levels of SiO_2_NPs could be seen as the culmination of these internal dynamic cascades leading to the ultimate cytotoxic impact. It reveals a complex regulation of IL-6 gene expression impacting cell viability after exposure. There may be a potential correlation between IL-6 gene expression reduction and a mechanism linked to cellular toxicity. However, at higher concentrations, an unknown mechanism increases IL-6 gene expression, reducing SiO_2_NPs’ cytotoxic effects. This mechanism combats cellular toxicity, allowing cells to function normally. At this stage, it is unclear if SiO_2_NPs alter the interleukin-6 expression through a signal from cell surface interaction or cell penetration, affecting intracellular pathways, requiring further investigation for clarification. These effects are concentration-dependent and not influenced by exposure times. As these effects contribute to respiratory complementation given the contribution of these effects to respiratory complications and lung-related diseases, it is imperative for workers exposed to nanomaterials, particularly in nano-industries, to implement respiratory safety measures tailored to their specific risks. The establishment of preventive standards for nano-workers is crucial to mitigate these health risks. Furthermore, this study underscores the necessity for additional research to elucidate the unidentified mechanisms underlying these health issues. By understanding these mechanisms, we can develop more effective preventive interventions against toxicity and identify contributing factors to respiratory problems. Even in the early stages of observation, the results presented in this article could advance basic research on comprehending the biological impacts of nanoparticles at the cellular level and have practical implications. Despite our research being limited by a tight budget, the important findings it unveils could urge authorities to prioritize the well-being of individuals, especially workers exposed to SiO_2_NPs, in order to enhance working conditions and standards by conducting similar studies on blood samples from industries dealing with these nanoparticles in the future.

## Data Availability

The datasets used and/or analyzed during the current study are available from the corresponding author upon reasonable request. Please contact the corresponding author for the data requests.
